# Phenotypical Characterization of Spleen Remodeling in Murine Experimental Visceral Leishmaniasis

**DOI:** 10.3389/fimmu.2020.00653

**Published:** 2020-04-15

**Authors:** Caroline Vilas Boas de Melo, Micely D'El-Rei Hermida, Bianca R. Mesquita, Jonathan L. M. Fontes, Jasper J. Koning, Manuela da Silva Solcà, Bruno B. Benevides, Girlândia B. S. Mota, Luiz A. R. Freitas, Reina E. Mebius, Washington L. C. dos-Santos

**Affiliations:** ^1^Laboratório de Patologia Estrutural e Molecular, Instituto Gonçalo Moniz, Fundação Oswaldo Cruz, Salvador, Brazil; ^2^Department of Molecular Cell Biology and Immunology, Amsterdam Infection and Immunity Institute, Amsterdam UMC, VU University Medical Center, Amsterdam, Netherlands

**Keywords:** visceral leishmaniasis, white pulp remodeling, spleen disorganization, lymphoid tissue inducer cells, spleen pathology

## Abstract

**Background:** Visceral leishmaniasis (VL) is caused by *Leishmania infantum* or *L. donovani* infection. One of the main problems related to this disease is the emergence of severe clinical forms with a lethality of 5–20%, even while under specific treatment. In humans and other species susceptible to fatal VL, such as dogs and hamsters, the disruption of splenic white pulp (WP) is accompanied by disease progression. Control of VL progression is seen in BALB/c mice, as evidenced by a mild clinical presentation and controlled parasite replication in the liver and spleen. In this study, we investigated the features involved in the morphological remodeling of splenic compartments associated with the control of VL progression to death.

**Methods:** We evaluated cohorts of BALB/c mice after 30, 60, and 90 days of infection by *L. infantum*. Spleen morphology, cell population subsets and cytokine production were studied in the spleen using flow- and histo-cytometry.

**Results:** Intraperitoneal infection with 10^8^ promastigotes of *L. infantum* led to progressive increases in spleen size at 60 and 90 days after infection. Splenomegaly was the only clinical sign of disease observed. At 30 days after infection, hyperplasia in the WP and decreased numbers of plasmacytoid dendritic cells were observed. The WP hyperplasia subsided at 60 days post-infection. However, the splenomegaly remained in association with increased numbers of macrophages, B and T lymphocytes and plasma cells. An increased number of lymphoid tissue inducer (LTi) cells was observed; these were distributed around the periarteriolar lymphoid sheath in control mice and scattered throughout the red pulp in the *Leishmania*-infected mice. After 90 days of infection, increased IL-6 and IFN-γ production was seen in the spleen, as well as higher frequencies of follicular and plasmacytoid dendritic cells.

**Conclusion:** The data presented herein emphasizes the potential role of spleen remodeling in the control of severe forms of VL and highlights features potentially involved in this process.

## Introduction

Visceral leishmaniasis (VL) is endemic in Central and South America, Asia, parts of Africa and the Mediterranean basin, with an estimated burden of 2.1 million DALY (disability adjusted life years) (1). It is a severe systemic parasitic disease caused by the protozoan *Leishmania donovani*, which affects humans, and *Leishmania infantum*, which affects humans and dogs. The current therapeutic approach with administration of glucantime, pentamidine or amphotericin is effective in most cases (2). However, VL is lethal in humans and dogs, even those under treatment. In Brazil, a ~37,209 individuals were affected by VL between 2001 and 2011, with a lethality rate of 6.8% (2,549 deaths) (3).

The main clinical signs of VL are weight loss, increased size of the liver and spleen, anemia, low platelet and neutrophil counts and increased susceptibility to bleeding and coinfections, leading to death (4, 5). The disease results from an inability of macrophages to kill the parasite (6). A complex signaling network of molecules produced by macrophages and T and B lymphocytes, with pro- and anti-inflammatory roles, produces an inflammatory status that is responsible for the many clinical manifestations of the disease without achieving control of parasite growth (5, 7). The bone marrow, liver and spleen are the most affected internal organs (8).

The spleen is a large lymphoid organ responsible for many physiological functions such as hemocatheresis and it is also responsible for immune surveillance against blood circulating pathogens (9). The spleen is organized into two main areas: white pulp (WP) and red pulp (RP). It is affected by *Leishmania* infection in all cases and during the entire course of the disease (10). Although the spleen compartments contain the crucial elements to effectively respond to *Leishmania* infection, in severe cases of disease, the spleen undergoes sequential changes of WP hyperplasia, atrophy and disruption (11). Spleen enlargement leads to hypersplenism syndrome with increased leukocyte and platelet retention and destruction of blood cells (12, 13).

In the late stages of severe VL, the WP is disrupted, germinal centers and mantle zones disappear, and lymphoid follicles are barely defined (14, 15). These changes are associated with decreased number of B lymphocytes, increased apoptosis of T lymphocytes, loss of follicular dendritic cells (FDCs), high parasite burden and change in the cytokine expression pattern (16–18). Loss of FDCs impairs production of CXCL13, a chemokine involved in B cell recruitment into the lymphoid follicles (19). Consequently, the B cells migrate to the RP where they differentiate into plasma cells (15), where overexpression of BAFF, APRIL, and CXCL12 contribute to an extended survival time of these cells (20). Progressive splenomegaly and remodeling of the splenic compartments are observed in experimental murine VL. Although extensive WP disruption was only observed after 60 days of infection, redistribution of marginal zone macrophages as well as RP vascular network remodeling were observed at 28 days post-infection (dpi) (11, 21, 22). The progressive lymphoid follicle depletion in murine VL was dependent on the initial inoculum size and the infection time (11, 23). Altogether, these alterations may interfere with memory T cell and B cell responses and contribute to an exacerbated and ineffective humoral immune response. The sequential cellular and molecular events leading to spleen compartment disorganization in VL still need to be elucidated. The fact that spleen disorganization is associated with more severe, sometimes, terminal disease, suggests that it plays a role in the progression of VL to a stage of no-response to current therapeutic approaches.

Lymphoid tissue inducer (LTi) cells are type 3 innate lymphoid cells (ILC3) characterized by expressing CCR6 with variable expression of CD4 (24, 25). In mice, these cells can be identified by expressing CD4 and not expressing lineage markers (e.g., CD3, B220, CD11c) (26). LTi cells interact with immune and stromal cells thereby promoting lymphoid tissue organogenesis such as lymph nodes and Peyer's patches (27–29). Although these cells are not critical for splenic WP development, they may provide early lymphotoxin signals in T cell areas and continue to play a role in WP organization in adult life (30, 31). For instance, LTi cells have been reported to participate in WP repair after injury caused by choriomeningitis virus infection (32). However, upon infection of mice with *L. donovani*, LTi cells appeared not to be crucial for splenic lymphoid tissue restoration induced by Sunitinib maleate (a tyrosine kinase inhibitor) (33).

In this study, we attempted to induce an extensive morphological disruption of the splenic WP in BALB/c mice upon prolonged infection with *L. infantum*, similar to that reported by Veress et al. (11). Our aim was to use this model to address the phenotypic cell changes leading to the profound disruption of the WP associated to severe chronic forms of VL. The data presented show that even heavily infected mice with a high parasite inoculum do not develop similar extensive spleen disorganization as seen in hamsters, dogs and humans. Furthermore, we observed changes in LTi cells distribution that may support a role played by these cells in the reorganization of splenic WP in the murine experimental model of infection with *L. infantum*.

## Materials and Methods

### Mice

BALB/c mice were obtained from the colony of IGM-FIOCRUZ. The animals were allocated into homogeneous experimental groups on the basis of sex, weight and age. They were kept in the experimental areas with food and water available *ad libitum* and under a controlled physiological regime of temperature and periods of light and dark.

### Parasites and Injection

*L. infantum* promastigotes (strain MHOM/BR2000/Merivaldo2) were maintained in passage in Golden Syrian hamsters and cultured *in vitro* until the stationary phase in complete Schneider medium (Schneider + 20% fetal bovine serum [FBS], Gibco, USA) in a B.O.D. incubator at 24°C. Mice were injected intraperitoneally (i.p.) at 6–8 weeks of age with either saline solution (control) or a parasite suspension of 10^7^ (first experiment) or 10^8^ (second experiment) promastigotes. Euthanasia was performed by overdose of anesthetics (10 mg cetamin + 1 mg xylazine/mL) at 30, 60, and 90 days post injection (dpi). The spleen was removed and divided into four fragments to perform flow cytometry, histology, histo-cytometry and qPCR. Clinical examination was performed according alterations of weight and hair loss, dehydration, skin lesions and splenomegaly. Evidence of infection was obtained by isolation and culture of parasites from the spleen (34). The parasite burden of the spleen was determined using qPCR as previously described (35). Anti-*Leishmania* antibody activity was detected by enzyme-linked immunosorbent assay (ELISA) in diluted (1:200) mouse serum as previously described (36).

### Isolation of Cells

For cell and cytokine flow cytometry analysis, the larger fragment of the spleen was weighed, then macerated in a cell strainer at 70 μm (BD Falcon) coupled to a 50 mL conical tube filled with phosphate-buffered saline (PBS). The cell suspension was centrifuged at 1,600 rpm/5 min/4°C. The cell-containing pellet was incubated in 2 mL of Red Blood Cells Lysis Buffer (BD Biosciences) for 2 min/37°C then centrifugated at 1,400 rpm/5 min/4°C. The cells were resuspended in 1 mL PBS and then the cell viability was assessed by trypan blue staining.

### Immunophenotyping of Leukocytes Using Flow Cytometry

Cell suspensions were incubated in FBS buffer with 5% mouse serum to block non-specific reactions. The cells were distributed in 100 μl of the suspension/well at a concentration of 2 × 10^6^ cells/mL. The following antibodies were used, all from BD Biosciences: fluorescein isothiocyanate (FITC)-conjugated anti-CD3 (17A2) and anti-CD11b (M1/70); phycoerythrin (PE)-conjugated anti-CD4 (GK1.5) and anti-B220/CD45R (30-F11); PE-Cy5-conjugated anti-F4/80 (BM8); Alexa Fluor (AF) 700-conjugated anti-CD19 (1D3); brilliant violet (BV) 421-conjugated anti-CD11c (N418) and anti-CD138 (281-2); BV 605-conjugated anti-MHC-II (I-A/I-E, M5/114.15.2); and brilliant blue (BB) 515-conjugated anti-CD23 (B3B4) and anti-CD93 (AA4.1) (Supplementary Table 1). Cells were incubated for 20 min with antibodies for the markers of interest and their respective control isotypes. Subsequently, cells were washed twice with PBS buffer. Cells were acquired using an LSRFortessa flow cytometer (BD Biosciences, USA). Gating strategy and data analysis were performed using FlowJo software (Tree Star Inc., California, USA).

### Splenocytes Stimulation Assay and Cytokine Mensuration by Flow Cytometry

Cell suspensions were transferred into 96-well plates in duplicate at 5 × 10^5^ cells. The plate was centrifuged at 2,000 g/10 s/4°C and the pellet was incubated with 100 μl of 50 μg/mL soluble *L. infantum* antigen (SLA) or 35 μl/mL concanavalin A (ConA) in Roswell Park Memorial Institute medium (RPMI) + 10% FBS. The plate was placed in a CO_2_ incubator at 37°C for 48 h. Then, it was centrifuged at 2,000 g/10 s/25°C and the supernatant was collected. A CBA Mouse Inflammation Kit Th1/Th2 (BD Biosciences) was used for measurement of IL-6, IL-10, MCP-1, IFN, TNF, and IL-12p70 in the supernatant of the pre-stimulated cells. Beads were acquired using an LSRFortessa cytometer (BD Biosciences) and analysis was performed using FlowJo software (Tree Star Inc., California, USA).

### Immunostaining and Image Acquisition

Spleen fragments were embedded in Tissue-Tek O.C.T. compound (Sakura Finetek, Japan Co., Ltd.) and frozen in the vapor of liquid nitrogen. Cryostat sections 4 μm thick were cut and placed onto negatively charged glass slides. The sections were air-dried and fixed in acetone for 10 min. Tissues were blocked for non-specific binding using 10% normal mouse serum for 15 min. Sections were incubated with specific antibodies (Supplementary Table 1) diluted in PBS + 2% newborn calf serum (NCBS) for the stromal cells panel and LTi cells panel. Sections were stained with fluorochrome-conjugated antibodies for 30 min, washed twice with PBS and finalized with mounting medium. For indirect antibody binding, sections were incubated with primary antibodies for 45 min, washed twice with PBS, and then incubated with secondary antibody for 30 min. The slides were washed in PBS and finalized with mounting medium. Negative controls were added per slide and treated with unconjugated antibodies and/or incubation buffer only. A Leica SP8 confocal microscope was used for image acquisition using LASX software (Leica Microsystems). Nine adjacent images were acquired in × 400 magnification into a mosaic to observe a larger area.

### Immunostaining of Stromal Cells

Stromal cells were immunostained using the following antibodies: anti-ER-TR9 (mAB derived from hybridoma cell culture supernatant) primary mouse antibody and AF 647-conjugated secondary antibody (Invitrogen); primary anti-mouse gp38 (anti-podoplanin 8.1.1, Developmental Studies Hybridoma Bank – DSHB –at University of Iowa, Iowa City, IA) antibody and AF 594-conjugated secondary antibody (Invitrogen); direct-labeled AF 488-conjugated anti-B220/CD45R (affinity-purified from hybridoma cell culture supernatant); AF 555-conjugated anti-MAdCAM (MECA79, affinity-purified from hybridoma cell culture supernatant); and BV 510-conjugated CD35 (8C12, BD Biosciences) (Supplementary Table 1). Images were acquired in 1024 × 1024 resolution and absolute intensity per area represented by each marker was measured by surface detailing by automatic software selection (Imaris, Oxford Instruments).

### Histo-cytometry of Lymphoid Tissue Inducer Cells

The phenotype of the LTi subsets was defined using a primary mouse antibody anti-IL-7Rα (A7R34, affinity-purified from hybridoma cell culture supernatant) and an AF 594-conjugated secondary antibody (Invitrogen) and/or AF 488-conjugated CD4 (GK1.5, affinity-purified from hybridoma cell culture supernatant). Cell lineages were immunostained with APC-conjugated CD11b (M1.70) and CD11c (N418) from eBioscience, and AF 647-conjugated B220 and CD3 (affinity-purified from hybridoma cell culture supernatant), detected by the same filter (Cy5) and emitted in the same range (660–665) as the signal of non-interest. The slides were washed in PBS, incubated with 4′,6-diamidine-2′-phenylindole dihydrochloride (DAPI) nuclear dye and finalized with mounting medium. Images were acquired in 2,048 × 2,048 resolution and an additional step of deconvolution of the image was performed using Huygens software (Scientific Volume Imaging). Strategy for image analysis was performed using Imaris software. Minimal fluorescence intensity was defined as the threshold for gating of co-localization and creation of a channel double positive for IL-7Rα and CD4. An arithmetic tool was used to subtract the fluorescence signal in two ways: (1) Lineage (CD11b/CD11c/CD3/B220) and CD4, to characterize the CD4^−^LTi subset (lineage^−^CD4^−^IL-7Rα^+^); and (2) Lineage (CD11b/CD11c/CD3/B220), to characterize the CD4^+^LTi subset (lineage^−^CD4^+^IL-7Rα^+^). The number of cells was manually assessed using a counting tool. Red dots were added over the CD4^−^IL-7Rα^+^LTi cells and green dots were added over the CD4^+^IL-7Rα^+^LTi cells to facilitate observation of the images. Dispersion of the CD4^+^IL-7Rα^+^ LTi cells was estimated by drawing the closest distance between these cells and the boundaries of the WP, morphometrically assessed using Image-Pro Plus version 6.0 software (MediaCybernetics, United States).

### Histopathology

Spleen fragments were fixed for 24–48 h in alcoholic acid formalin solution at room temperature. Spleen fragments were sliced to 3–4 mm thick, placed in histological processing cassettes and embedded in paraffin, then sectioned to 3–4 μm thick for staining with hematoxylin and eosin (H&E). The animal tissues were examined without prior knowledge of the groups, with the guidance of two pathologists (WLCdS and LARF). The intensity of inflammatory infiltrates, parasitism, fibrosis and cell degeneration were estimated. The spleen was further examined as described previously by Hermida et al. (37). Briefly, the degree of architectural organization of the spleen WP was classified as follows: well-organized when distinguishing the periarteriolar sheath from the lymphoid follicle, germinal center, mantle zone and marginal zone; slightly disorganized, which presents with atrophic or hyperplastic changes leading to a loss of definition of some WP regions, with their poorly individualized and distinct regions; and extensively disorganized, when the follicular structure is rarely distinct from the RP and T cell area (15, 37).

### Expression and Analysis of the Results

Numerical values are presented in tables or graphs representing absolute numbers, means, medians or percentages or fold changes relative to the control estimates as indicated. Comparisons of medians between control and infected groups were performed using Mann-Whitney test. For comparisons involving more than two groups ANOVA or Kruskal-Wallis tests was used and when recommended followed by Tukey's multiple comparison test. Time variations between groups were analyzed using ANOVA and Tukey's multiple comparison test. The threshold for statistical significance was set at a *p* < 0.05.

## Results

### General Characteristics of the Animals

**Figure 1:**
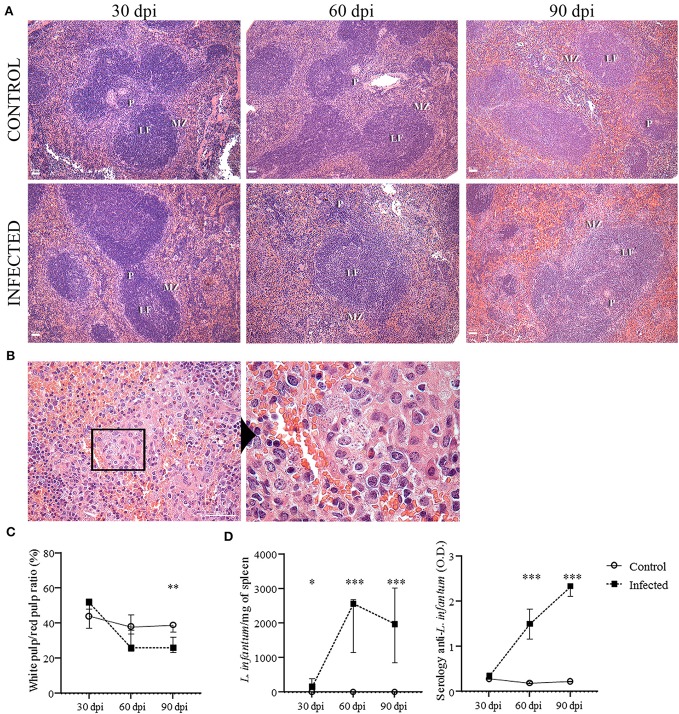
Histological changes of the spleen and evidence of infection. **(A)** Spleens of uninfected (control) and *L. infantum*-infected mice at 30, 60- and 90-days post-injection (dpi) (bars = 40 μm). MZ = marginal zone; LF = lymphoid follicle; PALS = periarteriolar lymphoid sheath. **(B**) Granuloma in the spleen of an infected mouse at 90 dpi (bar = 100 μm) evidencing amastigotes of L. infantum. **(C)** Morphometric estimation of white pulp (WP) and red pulp (RP) area represented in percentage of WP/RP ratio. Difference between control and infected mice (90 dpi), ^**^p = 0.006, Mann-Whitney test. **(D)** *L. infantum* per milligram of spleen of infected and control mice after 30 (^*^p < 0.05), 60 (^***^p < 0.001) and 90 (^***^p < 0.01) dpi (Kruskal-Wallis test). Optical density (O.D.) values for anti-*Leishmania* antibody activity in infected and control mice after 30, 60 (^***^p < 0.001) and 90 (^***^p < 0.001) dpi. The graphs represent the median and interquartile range (Kruskal-Wallis test).

Both experiments with inoculum of 10^7^ (presented as supplementary data) or 10^8^ presented similar results. However, the changes between infected and control groups were more expressive in the animals infected with 10^8^ promastigotes. The main characteristics of the mice infected with 10^8^ promastigotes are summarized in Table 1. As assessed by spleen culture and qPCR, evidence of infection by *L. infantum* was present in all infected mice, either infected with 10^7^ (Supplementary Table 2) or 10^8^ (Table 1 and Figure 1D) promastigotes and absent in control mice. Anti-*Leishmania* serology was negative in all control mice. Positive anti-*Leishmania* antibody detection was observed in 4/7 infected mice at 30 dpi and in all infected mice at 60 and 90 dpi (Table 1). A significant increase in the optical density (O.D.) for anti-*Leishmania* antibody activity was observed in infected mice after 60 dpi (1.5 [1.1–1.8]) and 90 dpi (2.3 [2.1–2.3]) in comparison to control mice (0.1 [0.1–0.2], 0.2 [0.2–0.2], *p* < 0.001, 60 and 90 dpi, respectively) (Figure 1D). Parasite burden progressively increased from 30 dpi (155.1 *L. infantum*/mg of spleen [151.8–391.2]) to 60 dpi (2,561 [1,153–2,690], *p* = 0.002) and to 90 dpi (1,978 [857.1–3,007], *p* = 0.01), and was negative in all control mice. Splenomegaly was characterized by an increase in the size and weight of the spleen and was observed at 60 and 90 dpi in mice infected with 10^8^ promastigotes (Table 1) but not in mice infected with 10^7^ promastigotes (Supplementary Table 2).

**Table 1:** Evidence of infection and clinical evaluation of the mice infected with 10^8^ promastigotes of *L. infantum*.

**Parameters**	**30 dpi**		**60 dpi**		**90 dpi**		***p*-value**
	**CT**	**INF**	**CT**	**INF**	**CT**	**INF**	
N (%)	7 (100)	7 (100)	7 (100)	7 (100)	7 (100)	6 (100)	
Evidence of infection by *L. infantum*							
Spleen culture	0	7 (100)	0	7 (100)	0	6 (100)	nt
Serology	0	4 (57)	0	7 (100)	0	6 (100)	nt
Clinical signs of VL							
Splenomegaly	0	0	0	7 (100)	0	6 (100)	nt
Spleen weight (g)^a^	0.1 ± 0.01	0.1 ± 0.004	0.1 ± 0.01	0.2 ± 0.05^b^	0.1 ± 0.01	0.5 ± 0.1^b^	<0.0001

CT, control group; INF, infected group;

a , spleen weight is expressed in average and standard deviation;

b *, statistical difference between control and infected groups per time point, t-test; nt, not tested*.

### Histological Evaluation of the Spleen

The splenic architecture was mainly preserved in all groups, although we observed mild disorganization of WP in 2/7 infected mice at 30 dpi and 1/6 infected mice at 90 dpi. This difference was not statistically significant (Figure 1A). There was a trend to a decrease in size of the WP area in the infected animals by 60 dpi. However, only at 90 dpi the area of the WP was significantly smaller in the infected compared to the control group (p = 0.006, Figure 1C). Hyperplasia of RP was observed in infected mice at 90 dpi (1 [0.7–1.2]) but not in the control mice (0 [0–0], *p* = 0.002]). Secondary lymphoid follicles with large germinal centers were more frequent in infected mice at 60 dpi (2 [1-2]) and 90 dpi (2 [1–3]) in comparison to the control groups (0 [−0.5–0.5], *p* = 0.01; 0 [−1–0], *p* = 0.001, 60 and 90 dpi, respectively). Lymphoid follicles were significantly increased at 90 dpi (2 [0.7–3], *p* = 0.03). Granulomas were only observed in infected mice at 60 dpi (1/7) and were more frequent and intense in infected mice at 90 dpi (Figure 1B) (6/6, *p* = 0.001). Only slight changes in the WP were observed in the animals infected with 10^7^ promastigotes of *L. infantum* without statistical difference (data not shown).

### Leukocyte Populations in the Spleen

**Figure 2:**
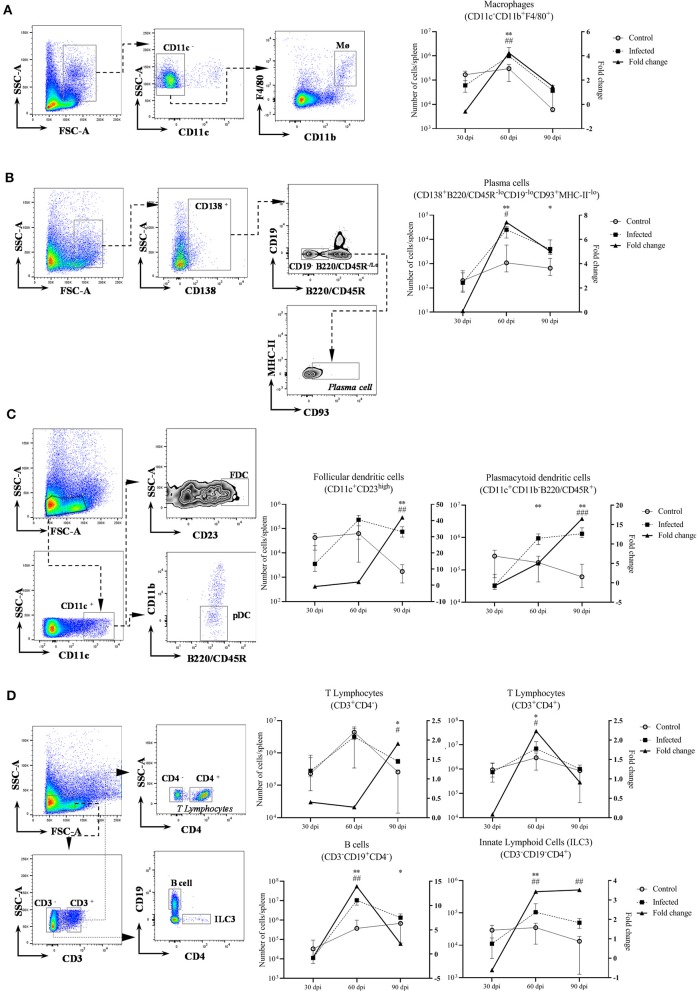
Leukocyte populations in the spleen of uninfected and 108 *Leishmania*-infected mice. Leukocyte populations at 30, 60 and 90 dpi, five mice per group (left Y: absolute number of cells per spleen; right Y: fold change of infected/control. Graphs represent median and interquartile range). ^*^ = statistical difference between control and infected groups per time point, Mann-Whitney test. ^#^= statistical difference between time points, ANOVA. **(A)** Macrophages (CD11c^−^CD11b^+^F4/80^+^), ^*^p = 0.007; ^#^*p* = 0.005, comparison between 30 dpi and 60 dpi; **(B)** plasma cells (CD138^+^B220/CD45R^−^loCD19^−^loCD93^+^MHC−II^−^lo), 60 dpi ^*^p = 0.007, 90 dpi ^*^p = 0.01; ^#^*p* = 0.04, comparison between 30 dpi and 60 dpi; **(C)** follicular dendritic cells (CD11c^+^CD23^high^), ^*^p = 0.007 and plasmacytoid dendritic cells (CD11b^−^CD11c^+^B220/CD45R^+^), ^*^p = 0.007; ^#^*p* < 0.002, comparison of 90 dpi with 30 dpi and 60 dpi; **(D)** T lymphocytes CD3^+^CD4^+^ and CD3^+^CD4^−^, ^*^p = 0.03; ^#^*p* = 0.01, comparison between 30 dpi and 60 dpi; B cells (CD3^−^CD19^+^CD4^−^) 60 dpi, ^*^p = 0.007 and 90 dpi, ^*^p = 0.03; ^#^*p* < 0.001, comparison of 60 dpi with 30 dpi and 90 dpi; innate lymphoid cells (CD3^−^CD19^−^CD4^+^), ^*^p = 0.007; ^#^*p* < 0.002, comparison of 30 dpi with 60 dpi and 90 dpi.

Significant alterations in the leukocyte populations were detected at 60 dpi (Figure 2). Infected mice at 60 dpi presented with an increased number of macrophages, plasma cells, plasmacytoid dendritic cells (pDC), CD4^+^ T lymphocytes and B cells. We also found an increased number of CD3^−^CD19^−^CD4^+^ cells in infected mice at 60 dpi, with a suggestive phenotype of ILC3 (Figure 2). The fold change of FDC, pDC and CD3^+^CD4^−^ T lymphocytes progressively increased over time of infection. The number of B cells, pDC and plasma cells remained increased to the last time point (90 dpi) in comparison to control mice. Similar trends were observed by 60 dpi in an independent experiment following a 10^7^ dose of *L. infantum*, including increased number of suggestive CD3^−^CD4^+^ ILC3 at 60 dpi in infected mice (Supplementary Figure 1).

### Cytokine Production by Splenocytes

**Figure 3:**
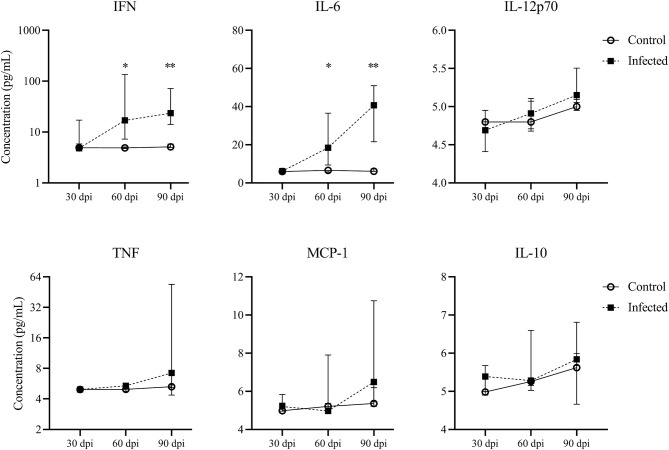
Cytokine production by splenocytes. Concentrations of IFNγ, IL-6, IL-12p70, TNF, MCP-1 and IL-10 in *Leishmania*-stimulated spleen cells of mice at 30, 60 and 90 dpi, five mice per group. Graphs represent the median and interquartile range. Statistical differences of concentrations of IFN at 60 (^*^p=0.03) and 90 dpi (^**^p = 0.007) and IL-6 at 60 dpi (^*^p = 0.02) and 90 dpi (^**^p = 0.007), Mann-Whitney test.

*Leishmania*-stimulated spleen cells in the infected mice produced higher concentrations of pro-inflammatory cytokine IFN at 60 dpi (16.9 pg/mL [7.2–135.4]) and 90 dpi (23.5 pg/mL [14–72]) than control mice (4.8 [4.7–5], *p* = 0.03; 5.1 [4.9–5.8], *p* = 0.007, 60 and 90 dpi, respectively) (Figure 3). A progressive increase in IL-6 concentration was observed in infected mice from 60 dpi (18.4 [9.3–36.6]) to 90 dpi (40 [21.5–50.9]) in comparison to control mice (6.5 [5.6–6.5], *p* = 0.02; 7.3 [6.1–7.5], *p* = 0.007, 60 and 90 dpi, respectively) (Figure 3). We did not observe statistical differences in MCP-1, TNF, IL-12p70 or IL-10 concentrations between the control and infected groups at any time point (Figure 3).

### Lymphoid Tissue Inducer Cells Distribution in Splenic Tissue

**Figure 4:**
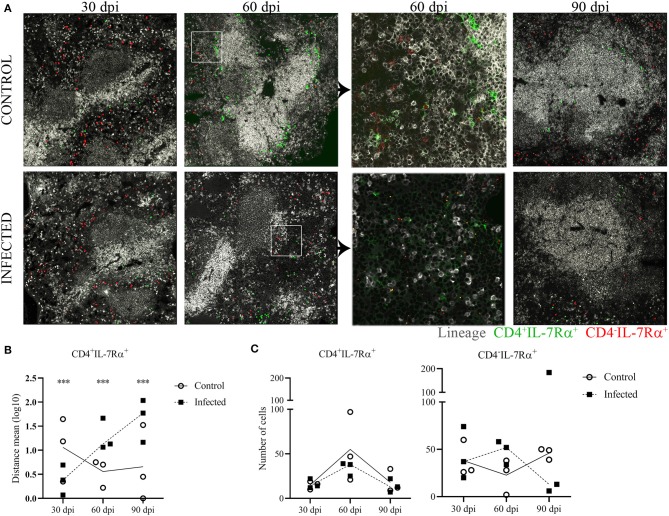
Lymphoid tissue inducer cells distribution in splenic tissue. Representative spleen confocal photomicrographs of *L. infantum*-infected and uninfected (control) mice after 30, 60 and 90 dpi, three mice per group. **(A)** Mosaic of nine pictures at × 400 magnification, bars = 50μm. Lineage B220/CD11b/CD11c/CD3 gray staining, CD4 green staining and IL-7Rα^+^ red staining. LineageCD4-IL-7Rα^+^ (red dots) represents CD4^−^ LTi cells and lineage^−^CD4^+^IL-7Rα^+^ (green dots) represents CD4^+^ LTi cells. **(B)** Log10 of distance means between the CD4^+^ LTi cells and the edges of the white pulp in the spleen of three mice per group after 30, 60 and 90 dpi (^***^p < 0.001), Kruskal-Wallis test. **(C)** Number of LTi cell subsets assessed by histo-cytometry of spleens of three mice per group after 30, 60 and 90 dpi.

Due to the finding of an increased number of likely ILC3-LTi cells by flow cytometry at 60 days of infection, we further investigated the distribution of these cells in the spleen. The phenotype for the CD4^−^ LTi subset was defined as lineage^−^CD4^−^IL-7Rα^+^ (red dots) and for the CD4^+^ LTi subset subtype as lineage^−^CD4^+^IL-7Rα^+^ (green dots). The CD4^−^ LTi cells were mainly located in the RP and similarly distributed in infected and uninfected animals (Figure 4A). In control mice, CD4^+^ LTi cells appeared mainly distributed around the periarteriolar lymphoid sheath (PALS) (Figure 4A, gray staining). However, CD4^+^ LTi cells were scattered in the RP in infected mice at 60 and 90 dpi as shown by the distance between the LTi cells and the edges of the WP (Figure 4B). Numeric differences of the LTi subsets were not evident in the histological analysis between infected and uninfected animals (Figure 4C).

### Stromal Cells Area Distribution in the Spleen

**Figure 5:**
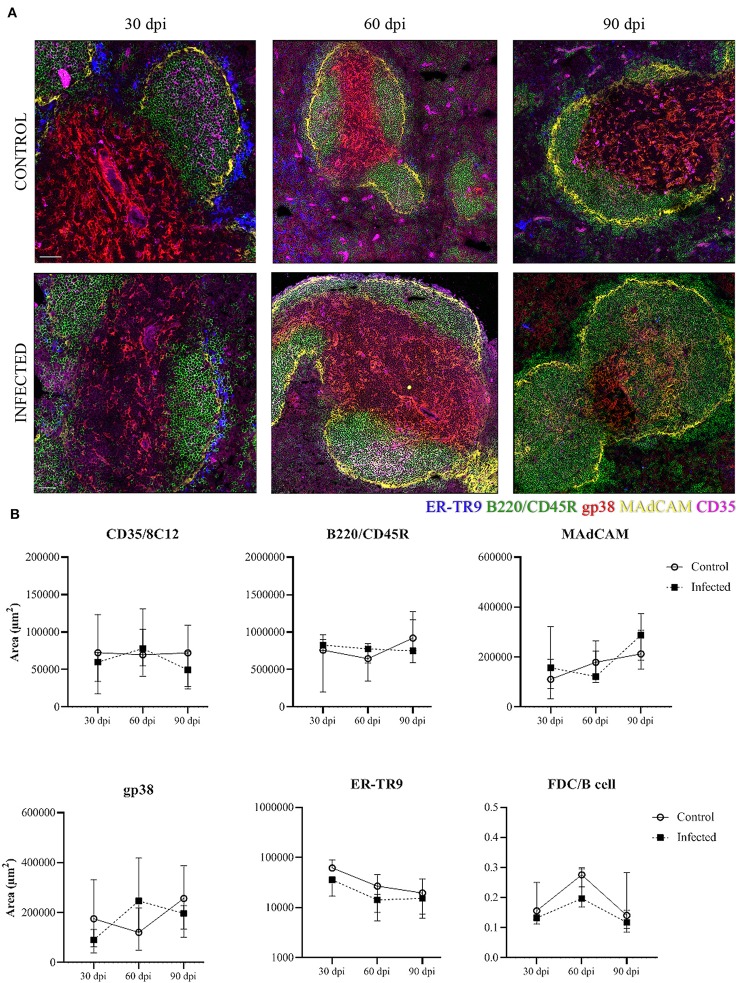
Stromal cells in the splenic tissue. Representative spleen confocal photomicrographs of *L. infantum*-infected and uninfected (control) mice after 30, 60, and 90 dpi. **(A)** Mosaic of nine pictures at × 400 magnification, bars = 50 μm. Stromal cell populations are represented by marginal reticular ER-TR9^+^ cells (blue), marginal reticular MAdCAM^+^ cells (yellow), B220/CD45R^+^ B cells (green) and follicular dendritic CD35^+^cells (magenta). **(B)** Quantification of positive area for stromal cell markers in the spleen after 30, 60, and 90 dpi. The graphs represent the median and interquartile range (Kruskal–Wallis test).

We analyzed the stromal cell distribution in the different compartments of the WP and the marginal zone. At 30 dpi a trend to a decrease of the marginal reticular ER-TR9^+^ cells (blue) area was observed in infected mice in comparison to control mice (Figure 5A). The ER-TR9^+^ cell area was similar between control and infected mice at 60 and 90 dpi (Figure 5B). For marginal reticular MAdCAM^+^ cells (yellow) the area was not different between uninfected and infected mice. In the lymphoid follicles, B220/CD45R^+^ B cell (green) area and FDC CD35^+^ cell (magenta) area were not different between groups (Figure 5), nor was the ratio FDC/B cells in the lymphoid follicle (Figure 5B). The area of fibroblastic reticular gp38^+^ cells in the PALS was not uniform across the different subjects (Figure 5).

## Discussion

In this work we studied the kinetics of spleen remodeling in late stages of chronic VL using an experimental model of infection in BALB/c mice. Early changes in cell populations and cytokines have been previously reported (16, 22, 23, 38). However, little is known about the progressive morphological changes that result in extensive disruption of the WP in the late stages of the disease. We observed that BALB/c mice infected with 10^8^ promastigotes of *L. infantum* developed a progressive follicle hyperplasia that became evident at 60 dpi. This spleen hyperplasia was associated with an increase in macrophages, B cells, plasma cells, follicular and plasmacytoid dendritic cells as well as ILC3-LTi cells. The distribution of LTi cells was also altered on the 60th day of infection. The WP area was significantly reduced at 90 dpi and Interferon gamma and IL-6 concentrations were consistently elevated in the spleen of the infected animals.

### Spleen Remodeling in the BALB/c Model of VL

Hyperplasia followed by atrophy and WP disruption is common in severe terminal leishmaniasis in some susceptible species such as humans and dogs (14, 39–41). Although hyperplasia and important cell changes took place in the mice in this study, only a decrease in WP area and a slight morphological disorganization of lymphoid tissue was observed, even though a large number of parasites were injected. Alterations in the splenic microenvironment have been reported upon natural *L. infantum*/*L. donovani* infection in human and dogs and in BALB/c mice and hamsters that were experimentally infected (11, 16, 22, 23, 38).

Substantial changes in cell populations in marginal zone and WP take place as early as 14 to 28 days of infection (21, 23, 33, 42). In this study we also observed changes in the ER-TR9^+^ stromal cell network in the marginal zone as well as WP hyperplasia associated with an increased number of macrophages, B cells, plasma cells, follicular and plasmacytoid dendritic cells and ILC3-LTi-like cells identified by flow cytometry from 30 to 90 dpi. A possible explanation for the late presentation of the splenic changes in our study is the intraperitoneal route used for infection. In most of the other studies the intravenous route was used (21–23). In this work, it seemed that some of the early alterations of the marginal zone and the WP were restored in later stages of the disease. It is not known how these initial and reversible changes may contribute to the late and potentially irreversible WP disruption. However, a decrease of lymphoid follicle size and lymphoid atrophy, following a period of lymphoid hyperplasia, seems to be dependent on the inoculum size and to remain through the subsequent course of the disease (11, 23). In fact, in this study, infection with 10^7^ promastigotes by intraperitoneal route only led to mild alterations in cell populations in the spleen (Supplementary Figure 1) and did not produce an increase in spleen size or weight. Although variations in cell counts occur in uninfected mice, the results of the two independent experiments with different inoculum sizes are similar. With a high parasite inoculum, important splenic changes were observed. Additionally, CD4^+^ LTi cells changed their distribution between 30 and 60 days of infection, as observed by histo-cytometry. The changes in the number and distribution of the cell populations, the peak of parasite burden and splenomegaly were all consistently present at 60 dpi, a key time-point in the progression of the disease. In fact, a complete reorganization of lymphoid tissue and vascular network may be involved in splenomegaly (22). In spite of all these changes a complete disruption of WP was not observed in this model of experimental murine VL.

These observations are consistent with the reported course of *L. infantum* infection in BALB/c mice, where splenomegaly and a paucity of clinical signs of VL are observed (22, 43). The parasite burden in the spleen reached a plateau at day 90 of infection, associated with a Th1 cytokine expression pattern. Together with the absence of a number of other clinical signs of disease apart from spleen enlargement, and only slight disorganization of the splenic structure, protective immunological signaling pathways may take place in murine *L. infantum* infection, as reviewed by Rodrigues et al. (44). The dynamics of ER-TR9^+^ network disruption and possible subsequent restoration, together with the LTi cell redistribution, suggests that control of spleen remodeling is also present in late stages of murine VL.

### Lymphoid Tissue Inducer Cells and Spleen Remodeling in BALB/c Mice VL

Evidence suggests that spleen lymphoid tissue organizers cells as well as LTi cells continue to play a role in WP maintenance in neonatal and adult life (30, 31). These cells persist around PALS and interfollicular areas (45) and may participate in WP regeneration after damage (46). In this study we show that the proportion of the CD4^+^ LTi subset was increased in the spleen between 60 to 90 dpi. These cells also were differentially distributed in the spleens of infected compared to uninfected mice. In most of the animals of the control group, CD4^+^ LTi cells were spotted surrounding the PALS, such as described for normal resting spleen (45). In the infected animals, CD4^+^ LTi cells showed no longer the same distribution, and were instead scattered in the RP at 60 and 90 dpi. Although the quantitative changes of CD4^+^ LTi cells observed by flow cytometry have been confirmed in two different experiments with different parasite inoculum, it was not possible to confirm these quantitative changes in upon histo-cytometry analysis. A possible explanation is that histo-cytometry (by using only 4 μm thick transversal sections of the spleen) may not be as sensitive as flow cytometry analysis (using 10^5^ dispersed cells) for detecting small variations in the number of cells represented in small quantities. However, histo-cytometry analysis allowed visualization of LTi subsets distribution in spleen compartments. Nevertheless, we cannot exclude the possibility that LTi cells play a role in the WP regeneration in severe VL. In fact, it has been shown that LTi cells still play a role in spleen embryogenesis although this role may not be critical (30). An increase in the proportion of LTi cells was also observed by Scandella et al. (32) in the spleen of mice infected with lymphocytic choriomeningitis virus. The increase in the proportion of these cells preceded the restoration of spleen histological structure. Vivier et al. (47) suggested that LTi cells also have a preventive role in lymphoid tissue damage. However, Dalton et al. (33) demonstrated using B6.Rorc^−/−^ → B6.CD45.1 chimeric LTi cell-deficient mice that WP regeneration in mice infected with *L. donovani* and treated with Sunitinib maleate could be independent of LTi cells. Although participation of LTi cells was not crucial to the WP regeneration in the work by Dalton et al. (33), their participation is not excluded in WP preservation in VL.

The data shown in this work together with the reported data in the literature on murine VL suggests that mice infected with *L. infantum* or *L. donovani* present some degree of spleen disorganization between 14 and 30 dpi evidenced by loss of marginal zone macrophages, disruption to both the FDC network in B cell follicles and transient disruption of the ER-TR9^+^ stromal cell network (16, 21). These alterations are followed by changes in the number and distribution of LTi cells and structural preservation of the WP up to 90 dpi.

Further studies are necessary to confirm the protective role played by LTi cells in preventing changes in the spleen upon VL and the mechanisms involved in this process.

## Data Availability Statement

The datasets generated for this study are available on request to the corresponding author.

## Ethics Statement

The animal study was reviewed and approved by Research Ethics Committee of Instituto Gonçalo Moniz (IGM-FIOCRUZ, license nos. 004/2013 and 017/2015).

## Author Contributions

CM and WS came up with the ideas for this experiment. CM, MH, BM, and JF planned and carried out the flow cytometry experiments. JK and RM planned the LTi and stromal cells panel. CM conducted the experiments and analysis. MS performed the qPCR for parasite burden. BB performed the morphometric analysis and obtained the serological evidence of infection. GM processed the tissues and prepared the slides. LF, WS, and CM performed the histopathological analysis. RM and WS supervised the project. CM and WS wrote the manuscript. All authors contributed to manuscript revision and read and approved the submitted version.

### Conflict of Interest

The authors declare that the research was conducted in the absence of any commercial or financial relationships that could be construed as a potential conflict of interest.

## Supplementary Material

Supplementary Figure 1 Leukocyte populations in the spleen of uninfected and 10^7^ *Leishmania*-infected mice. Leukocyte populations at 30, 60, 90, 120, and 150 dpi (left Y: absolute number of cells; right Y: fold change of infected/control. Graphs represent median and interquartile range). ^*^ = statistical difference between control and infected groups per time point, ANOVA. ^#^ = statistical difference between time points, ANOVA. Follicular dendritic cells (CD11c^+^CD23^high^), ^#^*p* = 0.03, comparison between 90 dpi and 120 dpi; plasmacytoid dendritic cells (CD11b^−^CD11c^+^B220/CD45R^+^), #*p* = 0.03, comparison of 90 dpi with 30 dpi; innate lymphoid cells (CD3^−^CD4^+^), ^*^*p* = 0.03; ^#^*p* < 0.001, comparison of 60 dpi with 30, 90, 120, and 150 dpi.

Supplementary Table 1 Anti-mouse antibodies for immunophenotyping.

Supplementary Table 2 Evidence of infection and clinical evaluation of the mice infected with 10^7^ promastigotes of *L. infantum*.
