# Supplementary figures and images for: Phenotypical Characterization of Spleen Remodeling in Murine Experimental Visceral Leishmaniasis
Source: Front Immunol. 2020 Apr 15;11:653. doi: 10.3389/fimmu.2020.00653 (PMC7174685; doi:10.3389/fimmu.2020.00653)

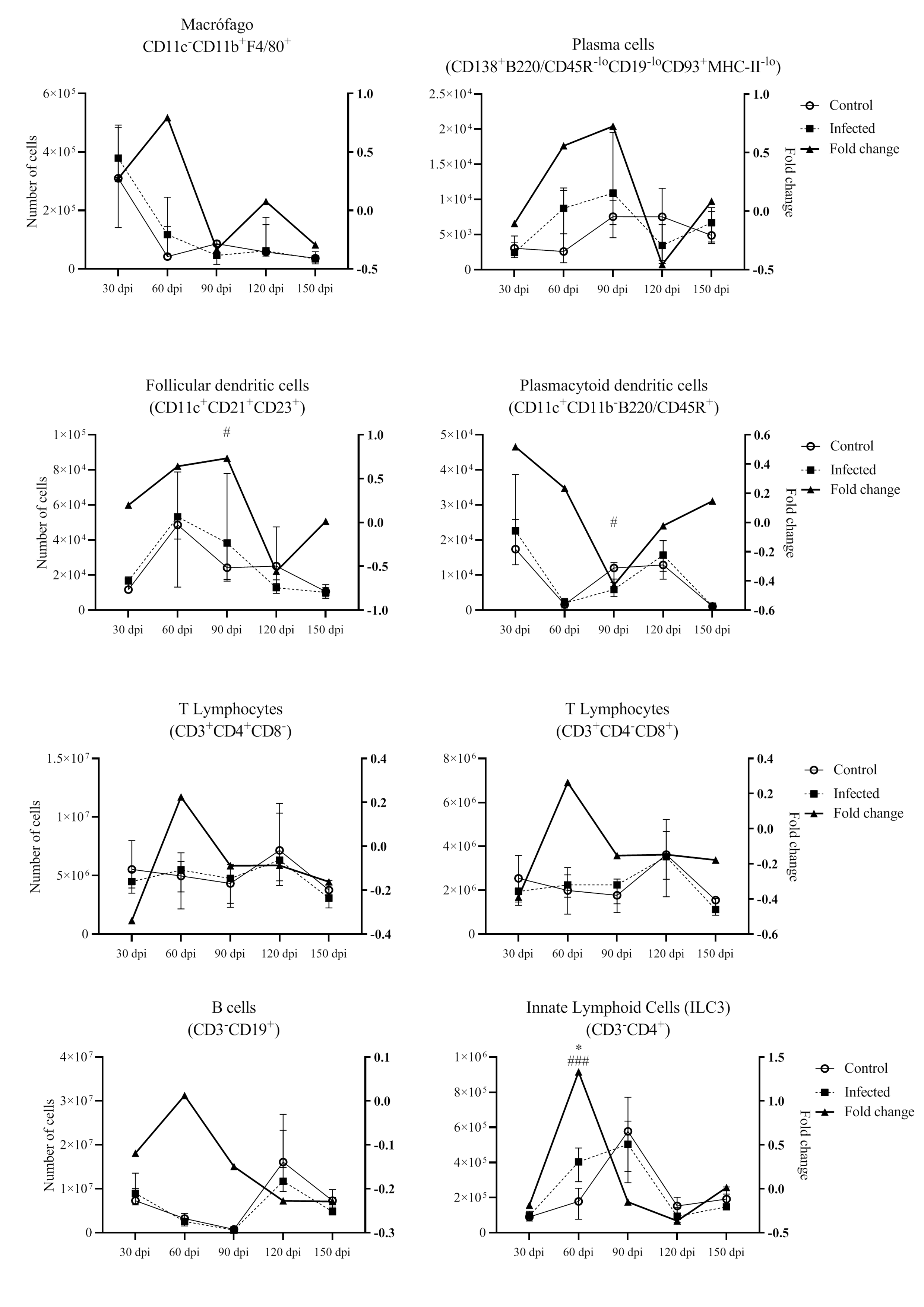

Supplement: Supplementary Figure 1 — Leukocyte populations in the spleen of uninfected and 107 Leishmania-infected mice. Leukocyte populations at 30, 60, 90, 120, and 150 dpi (left Y: absolute number of cells; right Y: fold change of infected/control. Graphs represent median and interquartile range). * = statistical difference between control and infected groups per time point, ANOVA. # = statistical difference between time points, ANOVA. Follicular dendritic cells (CD11c+CD23high), #p = 0.03, comparison between 90 dpi and 120 dpi; plasmacytoid dendritic cells (CD11b−CD11c+B220/CD45R+), #p = 0.03, comparison of 90 dpi with 30 dpi; innate lymphoid cells (CD3−CD4+), *p = 0.03; #p < 0.001, comparison of 60 dpi with 30, 90, 120, and 150 dpi. [file Image_1.TIF]
