# Supplementary material for: Phenotypical Characterization of Spleen Remodeling in Murine Experimental Visceral Leishmaniasis
Source: Front Immunol. 2020 Apr 15;11:653. doi: 10.3389/fimmu.2020.00653 (PMC7174685; doi:10.3389/fimmu.2020.00653)
Supplement: Supplementary Table 1 — Anti-mouse antibodies for immunophenotyping. [file Table_1.docx]

**Supplementary table 1:** Anti-mouse antibodies for immunophenotyping.

| Antibody | Fluorochrome | Supplier | Clone | Dilution |
| --- | --- | --- | --- | --- |
| CD3 | FITC | BD Biosciences 553061 | 17A2 | 1/100 |
| CD3 | AF647 | Affinity-purified from hybridoma cell culture supernatants and labeled with Alexa Fluor 647 | - | 1/50 |
| CD4 | PE | BD Biosciences 553730 | GK1.5 | 1/100 |
| CD4 | AF488 | Affinity-purified from hybridoma cell culture supernatants | GK1.5 | 1/200 |
| CD19 | AL700 | BD Biosciences 557958 | 1D3 | 1/200 |
| CD11b | FITC | BD Biosciences 01714D | M1/70 | 1/100 |
| CD11b | APC | eBioscience | MI/70 | 1/400 |
| CD11c | BV421 | BD Biosciences 565452 | N418 | 1/400 |
| CD11c | APC | eBioscience | N418 | 1/400 |
| F4/80 | PE-Cy5 | BD Biosciences 123112 | BM8 | 1/100 |
| CD23/FDC | BB515 | BD Biosciences 564637 | B3B4 | 1/200 |
| CD138 | BV421 | BD Biosciences 562610 | 281-2 | 1/400 |
| CD93 | BB515 | BD Biosciences 564700 | AA4.1 | 1/200 |
| B220 (CD45R) | PE | BD Biosciences 561087 | 30-F11 | 1/200 |
| B220 | AF488 | Affinity-purified from hybridoma cell culture supernatants | - | 1/800 |
| B220 | AF647 | Affinity-purified from hybridoma cell culture supernatants | - | 1/100 |
| CD19 | AL700 | BD Biosciences 557958 | 1D3 | 1/200 |
| MHC-II | BV605 | BD Biosciences 563413 | I-A/I-E M5/114.15.2 | 1/100 |
| CD35 | BV510 | BD Biosciences | 8C12 | 1/400 |
| MAdCAM | AF555 | Affinity-purified from hybridoma cell culture | MECA79 | 1/400 |
| ER-TR9 (rat anti-mouse) | Unlabeled | Derived from hybridoma cell culture supernatant | mAB | 1/10 |
| Anti-rat | AF647 | Invitrogen | - | 1/400 |
| IL-7Rα (rat anti-mouse) | Unlabeled | Affinity-purified from hybridoma cell culture supernatant | A7R34 | 1/1000 |
| Anti-rat | AF594 | Invitrogen | - | 1/400 |
| Gp38 (syrian hamster anti-mouse) | Unlabeled | Developmental Studies Hybridoma Bank – DSHB –at University of Iowa, Iowa City, IA | 8.1.1 | 1/50 |
| Anti-syrian hamster | AF594 | Invitrogen | - | 1/400 |
