# Supplementary material for: Phenotypical Characterization of Spleen Remodeling in Murine Experimental Visceral Leishmaniasis
Source: Front Immunol. 2020 Apr 15;11:653. doi: 10.3389/fimmu.2020.00653 (PMC7174685; doi:10.3389/fimmu.2020.00653)
Supplement: Supplementary Table 2 — Evidence of infection and clinical evaluation of the mice infected with 107 promastigotes of L. infantum. [file Table_2.docx]

**Supplementary table 2.** Evidence of infection and clinical evaluation of the mice infected with 10^7^ promastigotes of *L. infantum*.

| **Group** | **N** | **Spleen weight (g)^a^** | **Spleen culture** | **Parasite burden (*L.* infantum/g spleen)^b^** | **p value** |
| --- | --- | --- | --- | --- | --- |
| **30 dpi** |  |  |  |  |  |
| *Control* | 5 | 0,12 ± 0,02 | 0/5 | 0 [0-0] | ns |
| *Infected* | 6 | 0,1 ± 0,007 | 5/5 | **151.6 [12.1-520.2]*** | 0.005 |
| **60 dpi** |  |  |  |  |  |
| *Control* | 5 | 0,12 ± 0,03 | 0/5 | 0 [0-0] | ns |
| *Infected* | 5 | 0,15 ± 0,02 | 5/5 | 0 [0-312.6] | ns |
| **90 dpi** |  |  |  |  |  |
| *Control* | 5 | 0,15 ± 0,03 | 0/5 | 0 [0-0] | ns |
| *Infected* | 5 | 0,15 ± 0,02 | 5/5 | 6.8 [1.4-1468] | ns |
| **120 dpi** |  |  |  |  |  |
| *Control* | 4 | 0,14 ± 0,05 | 0/5 | 0 [0-0] | ns |
| *Infected* | 5 | 0,14 ± 0,04 | 3/5 | 92.4 [0-1027] | ns |
| **150 dpi** |  |  |  |  |  |
| *Control* | 5 | 0,10 ± 0,01 | 0/5 | 0 [0-0] | ns |
| *Infected* | 5 | 0,12 ± 0,01 | 5/5 | 6.7 [0-775.1] | ns |

Notes: ^a^ = spleen weight is expressed in average and standard deviation ^b^= parasite burden is expressed in median and interquartile range. *=statistical difference between control and infected mice at 30 days post injection (dpi), Kruskal-Wallis test. ns= not significant.
